# Response to Comment on “Artificial Intelligence Can Facilitate Application of Risk Stratification Algorithms to Bladder Cancer Patient Case Scenarios”

**DOI:** 10.1177/11795549251350233

**Published:** 2025-07-06

**Authors:** Max S Yudovich, Ahmad N Alzubaidi, Jay D Raman

We welcome the comments made by Daungsupawong and Wiwanitkit regarding our recent publication titled, “Artificial Intelligence can Facilitate Application of Risk Stratification Algorithms to Bladder Cancer Patient Case Scenarios.” Our study represents an early investigation into the capabilities of artificial intelligence (AI) with respect to the parsing and interpretation of clinical information. We agree that in practice, multiple sources of clinical data must be combined to assign risk for non-muscle-invasive bladder cancer (NMIBC). Per National Comprehensive Cancer Network (NCCN) guidelines, these include data from pathology reports (tumor grading), operative reports (tumor size), and clinical notes (tumor recurrences and BCG responses). We agree that further studies are warranted to investigate the practical applications of AI in NMIBC treatment. Our current use of deidentified patient information with artificial intelligence is limited by our institutional policy prohibiting such practice. HIPAA-compliant generative AI is achievable, though it requires significant resource investment by individual institutions.^1^

An exam-style approach was applied to the creation of the hypothetical NMIBC scenarios such that every tumor description would unambiguously represent an exact risk group of NMIBC. If such a scenario were an exam question, any urologist familiar with the NCCN guidelines would be capable of assigning the appropriate risk to the tumor. Each scenario was created based on the NCCN criteria for risk stratification of NMIBC, such that every combination of possible tumor characteristic was represented in the analysis.^2^ For example, Ta tumors can be small (less than or equal to 3 cm) or large (greater than 3 cm), low grade or high grade, and unifocal or multifocal. We aimed to have every combination of these features represented. Non-muscle-invasive bladder cancer is classified as low-, intermediate-, or high-risk (that is, categorical, rather than continuous). We agree that the terms “overestimation” and “underestimation” allow for ambiguity in a process that should be concrete. Therefore, better terms for our investigation would be “over-assignment” and “under-assignment” of risk by the large language model. For example, if GPT-4 assigned the high-risk designation to a low- or intermediate-risk tumor, this would represent over-assignment of risk by AI. Conversely, the assignment of low-risk designation to a high- or intermediate-risk tumor would constitute under-assignment of risk. We argue that under-assignment of risk is more dangerous to patient care than over-assignment. Under-assignment may result in missed diagnosis or tumor progression, whereas over-assignment may result in excess testing or financial toxicity.^3^

As clinical guidelines evolve, AI must adapt to use the latest information when assisting with clinical decisions. Newer guidelines should take precedence over older guidelines. An ideal medical AI model could reference a database of constantly updated guidelines without being trained on any specific outdated information. Within our study, this database of guidelines was included in the custom instructions for GPT-4. We agree that human supervision is mandatory in the implementation of AI in health care, particularly in the development of early integrations in health care. Physicians are adept at interpreting data with varying degrees of clarity and possess a high capacity for robustness with respect to missing or incomplete data. Further studies must be done to achieve similar reliability in AI for it to be a trustworthy tool in patient care.


Max S Yudovich, Ahmad N Alzubaidiand Jay D Raman
Milton S. Hershey Medical Center, Penn State Health, Hershey, PA, USA

